# Conflicts of Interest in “Throwaway” Dermatology Publications: Analysis of the Open Payments Database

**DOI:** 10.2196/30126

**Published:** 2021-07-22

**Authors:** Jorge Roman, David J Elpern

**Affiliations:** 1 The Ronald O. Perelman Department of Dermatology New York University Grossman School of Medicine New York, NY United States; 2 The Skin Clinic Williamstown, MA United States

**Keywords:** pharmaceutical industry, continuing medical education, dermatology, influence, payments, Open Payments database, publications, medical education, compensation, consulting, dermatologists

## Abstract

**Background:**

Dermatology journals, periodicals, editorials, and news magazines are influential resources that are not uniformly regulated and subject to influence from the pharmaceutical industry. This study evaluates industry payments to physician editorial board members of common dermatology publications, including “throwaway” publications.

**Objective:**

The aim of this study was to characterize the extent and nature of industry payments to editorial board members of different dermatologic publications in order to ascertain differences in payments between different types of publications.

**Methods:**

A list of editorial board members was compiled from a collection of clinical dermatology publications received over a 3-month period. Data from the Open Payments database from 2013 to 2019 were collected, and analysis of payments data was performed.

**Results:**

Ten publications were evaluated, and payments data for 466 physicians were analyzed. The total compensation across all years was US $75,622,369.64. Consulting, services other than consulting, and travel or lodging payments constituted most of the payments. A fraction of dermatologists received the majority of payments. The top payers were manufacturers of biologic medications. Payment amounts were higher for throwaway publications compared to peer-reviewed journals.

**Conclusions:**

Editorial board members of dermatology publications received substantial payments from the pharmaceutical industry. A minority of physicians receive the lion’s share of payments from industry. “Throwaway” publications have more financial conflict of interest than do peer-reviewed journals. The impact of these conflicts of interest on patient care, physicians' practice patterns, and patient perception of physicians is noteworthy.

## Introduction

Health care professionals across all specialties use a myriad of resources for staying up to date on the medical literature. Peer-reviewed journals are touted as the gold standard, while little attention has been given to the role of “throwaway” journals in keeping clinicians abreast of advances in the literature. Throwaway journals are characterized as publications that contain no original investigations, are provided free of charge, have a high advertisement to text ratio, are nonsociety publications, and are seldom peer reviewed [1]. Previous studies have shown that throwaway journals are more widely read than some peer-reviewed journals [1,2]. Throwaway journals are attractive to practicing clinicians given their ease of readability. The use of color, larger font size, graphics, and short summaries improve the appeal of throwaway journals to their readership [3].

Industry-physician interaction is common in all medical specialties, and dermatology is no exception [4]. Previous studies have examined conflicts of interests among authors of dermatology textbooks, dermatology patient advocacy organizations, dermatology clinical practice guideline authors, and clinical trials in dermatology [5-9]. Under the Physician Payment Sunshine Act, a part of the Affordable Care Act, payments and other transfers of value by manufacturers and group-purchasing organizations to physicians are reported to the Centers for Medicare and Medicaid Services. These payments are reported in categories including consulting, speaking fees, food, travel, and research [10].

Given the important role that journals play in the education and clinical practice of dermatologists, we sought to characterize the extent and nature of industry payments to editorial board members of different dermatologic publications, including the throwaway journals. Specifically, we examined the number, amount, and type of payments received, the companies that are contributing the payments, and physician-specific characteristics (sex, practice setting, fellowship training).

## Methods

To replicate a real-world scenario, publications related to clinical dermatology received by author JR (a dermatology resident) over a 3-month period were collected. All publication types, including peer-reviewed journals, non–peer-reviewed journals, and periodicals, such as news magazines and tabloids, were included for analysis. A list of editors was compiled by individually reviewing each publication. Editorial board members whose primary affiliation was outside of the United States and nonphysicians (ie, physician assistants and PhDs) were excluded from the study. Editor names were entered into the Open Payments database, and all payment data from 2013 to 2019 were collected. Physician-specific information on sex, practice setting, and training was collected via examination of professional information and biographies on individual practice websites. This study did not require approval by an institutional review board, as it did not contain human participants and used publicly available data. The Strengthening the Reporting of Observational Studies in Epidemiology (STROBE) reporting guidelines were used for this study [11]. Data analysis was completed using Excel version 16.41 (Microsoft Corporation). Descriptive statistics, including mean, median, IQR, and percentages were calculated. Median and IQR were used when appropriate as descriptors when there was a skewed distribution. The statistical significance of intergroup differences was tested by using an independent samples *t* test. A 2-tailed *P* value less than .05 was considered statistically significant.

## Results

Ultimately, 10 publications were evaluated, and 466 individual physicians were identified. The publications included 5 periodicals and 5 journals. The group consisted of mostly men (267/466, 57.3%). The proportions of physicians in academic and private practice settings were almost equal, with 51.9% (242/466) in private practice and 48.1% (224/466) in academic settings. However, of those in private practice, 67.8% (164/242) also held academic appointments. Furthermore, 21.0% (98/466) served on more than 1 editorial board. Further physician characteristics are shown in Table 1.

**Table 1:** Physician characteristics.

Physician characteristics and payment data	Value, n
Number of physicians	466
Physicians without payment data	52
Males	267
Osteopathic medicine	24
Mohs micrographic surgery	93
Dermatopathology	73
Pediatric dermatology	24
Private practice	242
Academic only	224
Serving on more than 1 editorial board	98

### Overall Payments

The total compensation across all years was US $75,622,369.64, and the total number of payments was 124,651. Of all physicians, 11.2% (52/466) had 0 payments reported. The median total industry payment was US $5334.69 (IQR US $331.23-89,837.74). This was higher than the median payment amount averaged from 2013 to 2019 for all US dermatologists (US $376.37) as well as the median payment for physicians across all specialties (US $1083.94) [12]. The median number of payments was 55 (IQR 4.3-295). This was also higher compared to the median number of payments for all dermatologists and US physicians across all specialties, with medians of 12 and 4, respectively. Apart from the period spanning 2017 to 2018, the total payment and number of payments increased yearly (Table 2).

Of the total payments (total amount), services other than consulting (US $31,392,593.02), consulting (US $22,201,879.20), and travel or lodging (US $8,071,910.76) payments constituted 81.54% (US $61,666,383/US $75,622,369.64) of payments (Multimedia Appendix 1). Associated research funding and research payments across all years totaled US $171,251,038.77 and US $17,618,505.85, respectively. The percentage of the cohort that received any kind of payments for associated research funding or research was small, at just 33.3% (155/466) and 25.5% (119/466), respectively. Of those who received payments, the median payment amount for associated research funding was US $204,284.45 (IQR US $39,659.32-960,049.20) and that for research payments was US $24,484.15 (IQR US $5017.50-144,941.78).

**Table 2:** Overall payment data^a^.

Payment data		Value
**All years**		
	General payments (number of payments)	75,622,369.64 (124,651)
	Research payments (number of payments)	17,618,505.85 (3325)
	Associated research funding (number of payments)	171,251,038.77 (22,076)
	Ownership and investment (number of payments)	26,144.08 (15)
	Median payment amount (IQR)	5334.69 (331.23-89,837.74)
	Median number of payments (IQR)	55 (4.3-295)
**2013-2019**		
	2013 total payments (number of payments)	30,64,126.60 (6462)
	2014 total payments (number of payments)	8,422,480.15 (17,094)
	2015 total payments (number of payments)	11,270,847.59 (19,093)
	2016 total payments (number of payments)	11,398,940.55 (20,021)
	2017 total payments (number of payments)	13,784,709.09 (21,225)
	2018 total payments (number of payments)	13,211,193.36 (20,232)
	2019 total payments (number of payments)	14,470,072.30 (20,815)

^a^Payment amounts are in US $.

### Academic Versus Private

Further analysis was performed after splitting the cohort by practice setting (academic vs private). Compared to those in academic settings, physicians in private practice had higher payments across all categories. The difference in payments was statistically significant for total general payments but not for research payments or associated research funding. Payment differences in the categories of services other than consulting, food and beverage, education, honoraria, and gifts were also found to be statistically significant (Table 3).

**Table 3:** Comparison of payments between physicians in academic versus private practice settings^a^.

Payment		Academic	Private	*P* value
Total general payments (number of payments)		26,210,268.14 (31,001)	49,412,101.50 (93,650)	*.03* ^b^
Total research payments (number of payments)		5,505,040.83 (1234)	12,113,465.02 (2091)	.09
Associated research funding (number of payments)		67,213,288.37 (7946)	104,037,750.40 (14,130)	.32
Median total general payments (IQR)		1,048.69 (94.46-19,575.68)	19,743.92 (1750.20-18,5248.15)	N/A^c^
Median number of total payments (IQR)		11 (1-63)	173 (41-463)	N/A
**Categories of payments**				
	Consulting (number of payments)	9,601,983.18 (2,981)	12,599,896.02 (3,838)	.44
	Services other than consulting (number of payments)	9,986,340.41 (3,545)	21,406,252.61 (7,810)	*.02*
	Travel and lodging (number of payments)	3,338,185.31 (8,634)	4,733,725.45 (13,608)	.31
	Food and beverage (number of payments)	807,956.04 (14,601)	2,505,852.32 (63,696)	*<.001*
	Education (number of payments)	66,933.34 (529)	192,825.57 (2,969)	*.01*
	Current or prospective ownership or investment interest (number of payments)	84,830.47 (4)	256,278.64 (2)	.52
	Honoraria (number of payments)	1,036,814.31 (288)	3,172,273.45 (829)	*.02*
	Faculty/speaker at an unaccredited/noncertified CME^d^ (number of payments)	777,345.36 (268)	1,463,257.45 (576)	.18
	Gift (number of payments)	2161.24 (126)	73,895.71 (401)	*.02*
	Grant (number of payments)	482,568.48 (18)	2,306,195.79 (58)	.12
	Faculty/speaker at an accredited CME (number of payments)	14,150 (6)	6310.50 (6)	.41
	Entertainment (number of payments)	0 (0)	101.85 (2)	.16
	Royalty or license (number of payments)	11,000 (1)	690,334.87 (4)	.32

^a^Payment amounts are in US $.

^b^Numbers in italics indicate statistical significance (*P*<.05).

^c^N/A: not applicable.

^d^CME: continuing medical education.

### Top Earners

The top 10% of physicians receiving payments collectively received US $56,060,893.28 which represented 74.13% (US $56,060,893.28/US $75,622,369.64) of the total payment amount for the entire study group. Moreover, 80.4% (37/46) of this subgroup received payments for research and associated research funding. In total, this cohort received US $102,076,943.74 in associated research funding and US $9,348,517.09 in research payments across all years, accounting for 59.61% (US $102,076,943.74/US $171,251,038.77) and 53.06% (US $9,348,517.09/US $17,618,505.85) of all payments in those categories, respectively. This group comprised mostly men (36/46, 78%), and the majority (33/46, 72%) worked in private practice. Of those in private practice, 79% (26/33) also held academic appointments, and 25 physicians served on more than 1 editorial board (mean 2.96; Table 4).

**Table 4:** Characteristics of the top 10% of physicians receiving payments^a^.

Characteristic	Value
Number of physicians	46
Total general payments (number of payments)	56,060,893.28 (65,644)
Total research payments (number of payments)	9,348,517.09 (1,832)
Total associated research funding (number of payments)	102,076,943.74 (12,715)
Males, n	36
Osteopathic medicine, n	2
Mohs micrographic surgery, n	5
Dermatopathology, n	3
Pediatric dermatology, n	3
Private practice, n	33
Academic only, n	13
Serving on more than 1 editorial board, n	25

^a^Payment amounts are in US $.

### Top Payers

The top 20 companies making payments were pharmaceutical manufacturers and combined paid US $64,774,389.91, representing 85.65% (US $64,774,389.91/US $75,622,369.64) of total disbursement. The majority of the companies were manufacturers of biologic medications (Table 5).

**Table 5:** Highest paying companies.

Company	Total general payments (US $)	Manufactured products
Abbvie	7,365,101.61	Adalimumab (Humira), risankizumab (Skyrizi), upadacitinib (Rinvoq)
Galderma	7,302,686.12	Hyaluronic acid gel filler (Restylane), abobotulinumtoxina (Dysport), poly-L-lactic acid filler (Sculptra), ivermectin cream (Soolantra), brimonidine topical gel (Mirvaso), adapalene and benzoyl peroxide (Epiduo)
Allergan (subsidiary of Abbvie)	5,993,810.99	Cross-linked hyaluronic acid filler (Juvederm), deoxycholic acid (Kybella), onabotulinumtoxina (Botox), cryolipolysis (Coolsculpting)
Bausch (Ortho dermatologics)	5,342,108.74	Brodalumab (Siliq), laser devices (via Solta)
Celgene	4,938,532.20	Apremilast (Otezla; sold in 2019)
Lilly	4,295,681.28	Ixekizumab (Taltz)
Regeneron	3,835,317.28	Dupilimab (Dupixent)
Novartis	3,599,007.79	Secukinumab (Cosentyx), ruxolitinib (Jakafi), omalizumab (Xolair)
Pfizer	3,435,221.32	Etanercept (Enbrel), tofacitinib (Xeljanz)
Genzyme	2,670,075.41	Dupilimab (Dupixent)
Janssen	2,500,056.99	Golimumab (Simponi), infliximab (Remicade), ustekinumab (Stelara), guselkumab (Tremfya)
Merz pharmaceuticals	2,232,056.79	Incobotulinumtoxina (Xeomin), calcium hydroxylapatite gel filler (Radiesse), hyaluronic acid filler (Belotero), intense focused ultrasound (Ultherapy), polidocanol (Asclera)
Leo pharma	1,967,161.84	Azelaic acid gel (Finacea), tacrolimus ointment (Protopic), topical vitamin D analogues
Almirall	1,938,517.92	Sarecycline (Seysara), dapsone gel (Aczone)
Bayer	1,521,718.32	Clotrimazole
Genentech	1,489,473.68	Vismodegib (Erivedge), rituximab (Rituxan), omalizumab (Xolair)
Amgen	1,276,055.07	Etanercept (Enbrel), apremilast (Otezla)
Sensus	1,071,990.39	Laser devices
Promius (subsidiary of Dr. Reddy's Laboratories)	1,012,017.41	Topical corticosteroids
Sun Pharma	987,798.76	Tildrakizumab-asmn (Ilumya)

### Individual Journal Analysis

Payment data for each individual publication was also performed. For simplicity, the publications were categorized into 2 groups, periodicals (including news magazines, tabloids, and editorials) and peer-reviewed journals. The average number of editorial board members for periodicals (mean 26.2) was lower than the average for peer-reviewed publications (mean 97.4). The averaged median payment amount (median US $113,877.02) to physicians on the editorial board members of the periodical publications was 3.5 times higher than to those on editorial boards of peer-reviewed publications (US $32,670.59). Associations with professional societies, patient advocacy organizations, access requirements, and other journal data are shown in Multimedia Appendix 2.

## Discussion

In this study we characterized payments from industry to editorial board members of clinical dermatology publications used as important resources in dermatology education and clinical practice. Our study shows that members of editorial boards of various types of publications have ties to industry. Exploration of this group demonstrates a facet of the medical industrial complex that pervades medicine. The data from this study showed that the remuneration received by editorial board members was on average 14 times higher compared to that received by dermatologists at large. Compensation for speaker fees, consulting, travel, and lodging made up most of the total payments. The 20 highest-paying manufacturers and most of the companies making payments to dermatologists belonged to the pharmaceutical industry. Dermatology as a specialty is a valued target for the pharmaceutical industry, being a relatively small field that treats several common and chronic conditions. Dermatologists are one of a few specialties that prescribe high-price biological medications. Of note, 11 of the top-20 paying companies in our study were manufacturers of biologic medications. Biologics for the treatment of psoriasis is a multibillion-dollar industry, representing some of the top-grossing medications in the world. Adalimumab (Humira) has been the top-selling drug for several years with over 19 billion dollars in global sales in 2019 alone [13]. Since gaining Food and Drug Administration approval for the treatment of adults with moderate to severe atopic dermatitis, dupilimab (Dupixent) sales have skyrocketed into the billions. The predominance of pharmaceutical payments in dermatology differs from other specialties such as orthopedic surgery, otolaryngology, and ophthalmology, in which device and diagnostic companies provide a greater amount of support [14-16].

Over the last several decades, the number of media by which clinicians acquire information to stay abreast of changes in their respective fields has increased. Historically, peer-reviewed journals were the mainstay source of information. With advancing technology, the market for resources that clinicians use for continuing medical education (CME) has expanded dramatically to embrace new formats, such as podcasts, webinars, virtual conferences, and social media applications (Instagram, Facebook, Twitter). Although most academic organizations have guidelines and policies to minimize or prevent conflicts of interests in publishing or the dissemination of information, the same cannot be said for other avenues that are independently sponsored or promoted. Today, most physicians, when faced with a surplus of journals, do not have the time to critically appraise each individual article. Instead of trying to grasp increasingly complex science or statistical methods, the more practical solution is to read summaries or condensations of educational material. This has paved the way for the “throwaway” journals. Throwaway journals usually do not contain any original investigations, have a high advertisement to text ratio, and are often provided free of charge, as they are funded by pharmaceutical companies. These journals are seldom peer-reviewed but are quite popular given their high readability [10]. The articles are frequently written by employees of the publication’s sponsoring companies and are seldom fact-checked by independent reviewers. They often include capsule summaries of conferences, journal article synopses, or therapeutic guides—all sandwiched between pharmaceutical ads. The information within throwaway journals is rarely antagonistic towards the pharmaceutical companies funding the journal [17]. Despite the popularity of throwaway journals and their direct role in clinician education, there is a dearth of research or discussion of throwaways in the literature. A PubMed search for throwaway journals returns just 8 results, with the last article being published in 2005 [18].

Distinguishing between a classic throwaway and a prototypical peer-reviewed journal has become increasingly difficult. For example, one of the periodicals (*DermWorld*) that was examined in this study was affiliated with a peer-reviewed journal (*Journal of the American Academy of Dermatology* [*JAAD*]). The median payment amounts (US $693.68 and US $1885.65, respectively) for both of these publications were the lowest (*DermWorld*) and second lowest (*JAAD*) in each of their respective groups. In contrast, the median payment amount for one of the peer-reviewed journals (US $146,159.48, *Journal of Clinical and Aesthetic Dermatology* [*JCAD*]) was 11 times greater than the next highest median payment for peer-reviewed journals (US $12,526.52, *Journal of Drugs in Dermatology*). *JCAD* is a peer-reviewed, PubMed-indexed publication platform wherein every article published is available as full text and free via PubMed but with costs covered by advertising and subscriptions. With the commercialization of medical literature and a move toward open-access type publishing, these hybrid types of journals are increasingly common. The bias is clear, and the conflicts of interest run deep. Affiliations with other entities, including patient advocacy organizations, specialty societies, and other groups that also receive financial support from the pharmaceutical industry, add another layer of complexity to the relationship.

It stands to reason that industry companies would select for well-known authorities and leaders in the field to provide their knowledge and expertise when evaluating their products. Historically, key opinion leaders (KOLs) have earned their positions by performing original research, discovering new therapies, and advancing the field. KOLs are well known in their respective fields, recognized as the authors of innovative journal articles, senior editors of major textbooks, specialty committee or leadership members, clinical practice guidelines authors, expert speakers at societal meetings, and institutional faculty leaders. Traditionally, the road to becoming a KOL involved years of research, teaching, and dissertation. However, currently some have asserted that becoming a KOL is more of a commercial enterprise carried out by the pharmaceutical industry and private KOL consulting firms [19,20]. A usual and effective method for industry to disseminate information is through peer advocacy [21]. This practice makes sense from a business standpoint, as KOLs are valuable figures that can lend expertise and credibility to new pharmaceuticals. Depending on the need, whether a company is looking to introduce a new product, rebrand a previous or newly reformulated product, or develop CME programs, KOLs can function as medical brand ambassadors to target specific audiences. The marketing value of KOLs is analogous to celebrity sponsorship deals in commercial ventures. The line between a trusted colleague sharing their knowledge and a salesperson selling a product is consequently blurred. In an unadulterated world, delivery of information by KOLs would be moral if the material were impartial and rooted in evidence-based medicine. However complete objectivity seems questionable when one party benefits so greatly. Industry offers many advantages to KOLs, including paid consultancy, participation in clinical trials, prestige in the eyes of peers, and opportunities for article authorship. The medical literature represents a useful avenue for industry to take advantage of the credibility and standing of KOLs [22]. The web of interaction is broad as evidenced by the activities of the top-paid dermatologists in our study. Many of the top earners serve on multiple editorial boards, hold dual private and academic appointments, and run a conglomerate of CME activities backed by industry for the purpose of influencing dermatologists at large. As examples, the highest earner received payments from 53 different companies, and one physician in the top 10% served on 6 editorial boards, including several of the peer-reviewed journals. A further 88.8% (414/466) of physicians in this study received payments from industry. This was higher than the percentage reported for dermatology textbook authors [6] (54.0%) and the 73.3% and 86% reported in the studies by Feng et al [4] and Checketts et al [23], respectively.

Historically, collaboration between physicians and the pharmaceutical industry has resulted in innovations and advancements in medicine. When conducted properly, the relationship between physicians and industry serves to advance the field of medicine as a whole with the ultimate goal of improving the lives of patients. However, the interests and commitments of physicians should deviate from those of industry. Where caring for patients is the primary responsibility of physicians, those in industry are chiefly concerned with their responsibility to their shareholders. As with any other business, the objectives of industry are geared towards profit. Industry engagement occurs so often that the practice has become a normalized component of physician education. This element of medical education has evolved over several decades and is so ubiquitous that many trainees and clinicians have become anesthetized to the practice. The fraternity of medicine is one in which new inductees observe their teachers and mentors giving industry-sponsored lectures, serving on industry advisory boards, and receiving industry funding for research [24]. These practices are so ingrained in our profession that participation is actually desirable for advancing academic careers or enhancing prestige. The “supportive” role of industry in medical education is ethically problematic.

Patients expect physicians to deliver effective, safe, and compassionate care based on evidence and best practices. As medicine is always changing, physicians must stay abreast of new therapeutics, devices, skills, and treatments. Establishing and upholding standards of competence is a responsibility of physicians to society. When these standards are perverted by industry, patients become unknowing victims of commerce. Over recent years, industry has played an increasingly direct role in physician education. The pharmaceutical industry’s exploitation of medicine is alive and well, flourishing through academic literature, commercial marketing, and compliant colleagues. Industry has become so intertwined with medicine that it shapes medical knowledge and opinion to suit its commercial needs. It has injected its presence into clinics, conferences, research, journals, and medical education. This relationship is not completely clandestine. Funding from industry supports research grants, clinical trials, and educational programs. As physicians we need to be aware of how industry influences the information required to care for our patients. To suit the needs of industry, promotion and marketing sway the independence of information presented to clinicians. The quality and integrity of clinician education is paramount in maintaining the public’s trust in our profession. In order to maintain the standards of postgraduate professional education, the relationship between industry and accredited education must be made transparent.

Editorial board members of dermatology publications received substantial payments from the pharmaceutical industry. A minority of physicians receive the lion’s share of payments from industry. Throwaway publications have more financial conflicts of interest than do peer-reviewed journals. The impact of these conflicts of interest on patient care, physicians’ practice patterns, and patient perception of physicians is noteworthy.

## Appendix

Multimedia Appendix 1 Categories of payments.

Multimedia Appendix 2 Individual journal characteristics and payments data.

## Abbreviations

CME: continuing medical education
JAAD: *Journal of the American Academy of Dermatology*
JCAD: *Journal of Clinical and Aesthetic Dermatology*
KOL: key opinion leaders
STROBE: Strengthening the Reporting of Observational Studies in Epidemiology
