# Supplementary material for: Conflicts of Interest in “Throwaway” Dermatology Publications: Analysis of the Open Payments Database
Source: JMIR Dermatol. 2021 Jul 22;4(2):e30126. doi: 10.2196/30126 (PMC10334964; doi:10.2196/30126)
Supplement: Multimedia Appendix 1 [file derma_v4i2e30126_app1.pdf]

Table 1. Categories of payments.

Categories of payments	
Services other than consulting (number of payments)	$31,392,593.02 (11,355)
Consulting (number of payments)	$22,201,879.20 (6,819)
Travel and lodging (number of payments)	$8,071,910.76 (22,242)
Honoraria (number of payments)	$4,209,087.76 (1,117)
Food and beverage (number of payments)	$3,313,808.36 (78,297)
Grant (number of payments)	$2,788,764.27 (76)
Faculty/speaker at an unaccredited/non-certified CME (number of payments)	$2,240,602.81 (844)
Royalty or license (number of payments)	$701,334.87 (5)
Current or prospective ownership or investment interest (number of payments)	$341,109.11 (6)
Education (number of payments)	$259,758.91 (3,498)
Gift (number of payments)	$76,056.95 (527)
Faculty/speaker at an accredited CME (number of payments)	$20,460.50 (12)
Entertainment (number of payments)	$101.85 (2)
