# Supplementary material for: Conflicts of Interest in “Throwaway” Dermatology Publications: Analysis of the Open Payments Database
Source: JMIR Dermatol. 2021 Jul 22;4(2):e30126. doi: 10.2196/30126 (PMC10334964; doi:10.2196/30126)
Supplement: Multimedia Appendix 2 [file derma_v4i2e30126_app2.pdf]

Table 2. Individual journal characteristics and payments data.

**Journal characteristics**	
**Dermatology News**	
Category	Periodical
Access	Free
Number of editorial board members	23
Members serving on more than 1 editorial board (%)	8 (34.8)
Total general payments (number of payments)	$4,459,080.96 (7,707)
Total research payments (number of payments)	$1,007,120.56 (319)
Total associated research funding (number of payments)	18,916,442.59 (2,114)
Median total general payments (number of payments)	$41,326.01 (187)
**The Dermatologist**	
Category	Periodical
Access	Free
Associations	National Psoriasis Foundation, The National Eczema Association, The National Rosacea Society, The Skin Cancer Foundation
Number of editorial board members	32
Members serving on more than 1 editorial board (%)	25 (78.1)
Total general payments (number of payments)	$20,393,699.41 (23,285.00)
Total research payments (number of payments)	$6,170,311.33 (958)
Total associated research funding (number of payments)	$35,322,265.41 (5,157)
Median total general payments (number of payments)	$ 198,453.28 (383)
**Practical Dermatology**	
Category	Periodical
Access	Free
Number of editorial board members	47
Members serving on more than 1 editorial board (%)	28 (59.6)
Total general payments (number of payments)	$23,157,228.57 (33,383)
Total research payments (number of payments)	$4,899,282.62 (687)
Total associated research funding (number of payments)	$33,955,728.77 (3,880)
Median total general payments (number of payments)	$244,372.97 (428)
**Dermatology Times**	
Category	Periodical
Access	Free
Number of editorial board members	12
Members serving on more than 1 editorial board (%)	9 (75)
Total general payments (number of payments)	$3,660,767.34 (6,348.00)
Total research payments (number of payments)	$2,155,154.69 (462)

Total associated research funding (number of payments)	$18,325,820.76 (2,546)
Median total general payments (number of payments)	$84,539.15 (380)
**DermWorld**	
Category	Periodical (affiliated with peer-reviewed journal)
Access	Paid subscription
Associations	American Academy of Dermatology
Number of editorial board members	17
Members serving on more than 1 editorial board (%)	5 (29.4)
Total general payments (number of payments)	$284,468.87 (1,100)
Total research payments (number of payments)	$72 (1)
Total associated research funding (number of payments)	$115,985.54 (49)
Median total general payments (number of payments)	$693.68 (5)
**Cutis**	
Category	Journal reviewed by editorial board members, indexed in MEDLINE®
Access	Free
Associations	Association of Military Dermatologists, Skin of Color Society, Society of Dermatology Hospitalists
Number of editorial board members	114
Members serving on more than 1 editorial board (%)	40 (35.1)
Total general payments (number of payments)	$18,590,790.23 (26,960)
Total research payments (number of payments)	$5,872,727.48 (1,164)
Total associated research funding (number of payments)	$43,050,936.87 (4,399)
Median total general payments (number of payments)	$2,628.25 (37)
**JAMA Dermatology**	
Category	Peer-reviewed journal indexed with MEDLINE®
Access	Paid subscription
Number of editorial board members	25
Members serving on more than 1 editorial board (%)	7 (28)
Total general payments (number of payments)	$1,712,629.65 (2,366)
Total research payments (number of payments)	$115,610.91 (111)
Total associated research funding (number of payments)	$3,433,872.97 (548)
Median total general payments (number of payments)	$153.04 (2)
**Journal of the American Academy of Dermatology**	
Category	Peer-reviewed journal indexed with MEDLINE®
Access	Paid subscription
Number of editorial board members	127
Members serving on more than 1 editorial board (%)	35 (27.6)

Total general payments (number of payments)	$15,435,274.85 (22,313)
Total research payments (number of payments)	$3,275,731.90 (794)
Total associated research funding (number of payments)	$36,199,238.01 (5,599)
Median total general payments (number of payments)	$1,885.65 (18)
**Journal of Clinical and Aesthetic Dermatology**	
Category	Peer-reviewed journal indexed with PubMed but not indexed with MEDLINE®
Access	Free
Associations	American Cutaneous Oncology Society, The Dermatologic & Aesthetic Surgery International League, American Acne & Rosacea Society
Number of editorial board members	91
Members serving on more than 1 editorial board (%)	52 (57.1)
Total general payments (number of payments)	$39,619,853.99 (60,310)
Total research payments (number of payments)	$12,024,264.82 (1,809)
Total associated research funding (number of payments)	$84,388,373.29 (11,295)
Median total general payments (number of payments)	$146,159.48 (412)
**Journal of Drugs in Dermatology**	
Category	Peer-reviewed journal indexed with PubMed and MEDLINE®
Access	Free for residents and fellows, complimentary 3-year subscription for dermatologists
Associations	International Society of Dermatologic Surgery, Skin of Color Seminar Series, Orlando Dermatology Aesthetic and Clinical Conference
Number of editorial board members	130
Members serving on more than 1 editorial board (%)	41 (31.5)
Total general payments (number of payments)	$25,071,730.66 (37,238)
Total research payments (number of payments)	$9,172,439.26 (1,272)
Total associated research funding (number of payments)	$55,232,215.29 (6,874)
Median total general payments (number of payments)	$12,526.52 (105)
